# Offspring of Obese Dams Exhibit Sex-Differences in Pancreatic Heparan Sulfate Glycosaminoglycans and Islet Insulin Secretion

**DOI:** 10.3389/fendo.2021.658439

**Published:** 2021-05-24

**Authors:** Jose Casasnovas, Christopher Luke Damron, James Jarrell, Kara S. Orr, Robert N. Bone, Stephanie Archer-Hartmann, Parastoo Azadi, Kok Lim Kua

**Affiliations:** ^1^ Department of Pediatrics, Indiana University School of Medicine, Indianapolis, IN, United States; ^2^ Complex Carbohydrate Research Center, University of Georgia, Athens, GA, United States

**Keywords:** islet insulin secretion, offspring of obese mothers, developmental origin of adult health and diseases, maternal obesity, heparan sulfate glycosaminoglycan

## Abstract

Offspring of obese mothers suffer higher risks of type 2 diabetes due to increased adiposity and decreased β cell function. To date, the sex-differences in offspring islet insulin secretion during early life has not been evaluated extensively, particularly prior to weaning at postnatal day 21 (P21). To determine the role of maternal obesity on offspring islet insulin secretion, C57BL/6J female dams were fed chow or western diet from 4 weeks prior to mating to induce maternal obesity. First, offspring of chow-fed and obese dams were evaluated on postnatal day 21 (P21) prior to weaning for body composition, glucose and insulin tolerance, and islet phasic insulin-secretion. Compared to same-sex controls, both male and female P21 offspring born to obese dams (MatOb) had higher body adiposity and exhibited sex-specific differences in glucose tolerance and insulin secretion. The male MatOb offspring developed the highest extent of glucose intolerance and lowest glucose-induced insulin secretion. In contrast, P21 female offspring of obese dams had unimpaired insulin secretion. Using SAX-HPLC, we found that male MatOb had a decrease in pancreatic heparan sulfate glycosaminoglycan, which is a macromolecule critical for islet health. Notably, 8-weeks-old offspring of obese dams continued to exhibit a similar pattern of sex-differences in glucose intolerance and decreased islet insulin secretion. Overall, our study suggests that maternal obesity induces sex-specific changes to pancreatic HSG in offspring and a lasting effect on offspring insulin secretion, leading to the sex-differences in glucose intolerance.

## Introduction

In the United States, over one million infants are born to obese mothers annually (1–4). At adulthood, offspring born to obese mothers have a higher risk of developing obesity (5–9) and insulin resistance (10–12). Recently, offspring of obese mothers were found to have a 3.5 fold higher risk of developing type 2 diabetes at young adulthood (13). The development of diabetes indicates a mismatch between an increased insulin demand secondary to insulin resistance and insufficient islet β cell insulin secretion. To date, the sex-specific impact of maternal obesity on pancreatic β cell insulin secretion in offspring of obese mothers is increasingly recognized, but still largely not well characterized.

Human and animal studies have demonstrated the important role of in-utero and early postnatal environments in modulating offspring islet insulin secretion (13–16). This concept of the developmental origin of health and diseases (DOHaD) was first described by Dr. Barker and Dr. Osmond, where adults that experienced growth restriction during fetal life were found to have a higher rate of cardiovascular diseases during adulthood (17–19). These offspring were also more likely to develop obesity and glucose intolerance (20, 21), and this increased risk was attributed to the early pre- and postnatal exposure to an altered nutritional environment (22). Further, there is compelling evidence of sex-differences in the risks of offspring developing obesity or glucose intolerance (23, 24). For example, studies in children born to diabetic mothers identified sex-specific differences in the risk of developing of glucose intolerance (23). Similar to the offspring of diabetic mothers, young adults born to obese mothers have a higher tendency to become obese (8, 24) and have lower insulin sensitivity (5, 25). However, the sex-specific impact of maternal obesity exposure on insulin secretion in human offspring has not been studied extensively. Therefore, animal models have been used to delineate the specific impact of maternal obesity on offspring islet insulin secretion (16, 26, 27).

Diet-induced obesity is a commonly used approach to model maternal obesity (16). While varying in diet compositions and duration of exposure, many studies have reported relatively consistent findings where offspring born to dams with pregestational obesity have hypertension, endothelial dysfunction (28–30), increased adiposity (31, 32), and decreased insulin sensitivity (31). In regard to pancreatic islet β cell health, a number of preclinical studies reported an altered islet architecture and β cell mass in offspring of obese dams (26, 33–36). In contrast, only two studies evaluated insulin secretion of both male and female young adult (8-weeks) offspring (16, 27). These studies found that male offspring of obese dams have a lower capability of maintaining insulin secretion (16, 27). Interestingly, Zambrano et al. suggested the presence of islet insulin secretory defects in offspring at a much younger age (5 weeks) (26). To date, the sex-differences in offspring islet insulin secretion before weaning at postnatal day 21 (P21) has not been evaluated. Notably, weaning to regular chow diet has been identified as a critical trigger inducing the maturation of pancreatic beta cell insulin secretion (37, 38). Therefore, it is essential to characterize sex-specific differences in offspring islet insulin secretion before weaning to identify the primary islet insulin secretory defects and mechanistic target(s) programmed by maternal obesity.

Finally, we will define the impact of maternal obesity on offspring pancreatic heparan sulfate glycosaminoglycans (HSG). HSG consists of a heparan sulfate core protein linked to a linear polysaccharide that is formed by repeating disaccharide units. Preclinical studies using genetic knock out animals also showed that the disruption in pancreatic islet HSG resulted in glucose intolerance and decreased islet insulin secretion (39). In humans, the decrease in islet HSG has been reported as an indicator for type 1 diabetes progression (40). Unfortunately, no studies have evaluated the impact of maternal obesity on offspring pancreatic HSG. Therefore, we aimed to delineate the role of maternal obesity on offspring pancreatic islet insulin secretion and pancreatic HSG. We hypothesize that the exposure to maternal obesity programs offspring islet insulin secretion and pancreatic HSG in a sex-specific manner.

## Materials and Methods

### Animals

All procedures conformed to the regulations of the Animal Welfare Act and the National Institutes of Health Guide for the Care and Use of Laboratory Animals, and were approved by the Indiana University School of Medicine Institutional Animal Care and Use Committee (Protocol #19161). Animals were housed in a temperature controlled, 12-hour light-dark cycled animal care facility with free access to water and food.

### Diet Induced Maternal Obesity

Four to five-week-old C57BL/6J female mice were purchased from Jackson Laboratory (Bar Harbor, ME) and randomly assigned to regular chow (2018SX, Envigo, IN) as a control, or western style diet (TD.88137, Envigo, IN) to induce maternal obesity (see **Supplemental Table 1** for diet composition). The chow and selected western diet were phytoestrogen-reduced to avoid the effects of exogenous estrogen (41). The female mice were maintained on the same diet for four weeks prior to mating, and through pregnancy and the lactating period. After birth, the litters were culled to 6-8 pups per litter. At postnatal day 21 (P21), offspring were subjected to *in-vivo* metabolic evaluation and euthanized, or weaned to a regular chow diet and evaluated at 8-weeks old (8W). Con and MatOb offspring of the same sex were weaned to the same cage. Female offspring were not exposed to males upon weaning, and the estrous cycle in female offspring were not synchronized/evaluated prior to metabolic studies to minimize stress.

### *In-Vivo* Metabolic Evaluations of Dams and Offspring

The body composition of female dams, P21 offspring, and 8W young adult offspring were measured using EchoMRI (EchoMRI LLC, Houston, TX) (14). Intraperitoneal glucose tolerance testing (GTT) and intraperitoneal insulin tolerance testing (ITT) were performed as published previously (14, 42). Briefly, animals were fasted for 5-6 hours prior to GTT, and 2 hours prior to ITT. The lower fasting time for ITT was to avoid the subsequent decrease in blood glucose level below 80 mg/dL, possibly triggering counter regulatory response. For GTT, 1 g/kg of glucose was injected intraperitoneally and blood was collected from animals *via* tail tip biopsy at 0, 10, 20, 30, 60, and 90-minute time points. For ITT, 0.75 U/kg of Humulin R (Eli Lilly, IN) was administered intraperitoneally and blood glucose was measured at 0, 15, 30, 45, and 60-minute time points. Animal blood glucose levels were measured with an Alphatrak Glucometer (Zoetis, NJ). Glucose area under curve (AUC) was calculated using the trapezoidal rule.

### Isolation of Pancreatic Heparan Sulfate Glycosaminoglycans (GAGs)

Whole GAGs were isolated from pancreata using methods described previously (43–45). Briefly, the samples were homogenized, defatted in acetone over two 24-hour periods at 4°C, and then subsequently digested in solution containing 2 mL 0.1 M Tris-HCl, pH 8.0, 2 mM CaCl_2_, 1% Triton X-100, and pronase (Roche) 0.8 mg/mL at 55°C. After 48 hours, the enzyme was inactivated by heating to 100°C for 15 min. The buffer was adjusted to 2 mM MgCl^2^, benzonase (Sigma; 100 mU) was added, and the sample was incubated for 2 h at 37°C. After inactivation of the enzyme (15 min, 100°C) any undigested material was removed by centrifugation for 1 h at 4000 g. The supernatant was applied to a DEAE-Sepharose (GE Healthcare; 2mL resin), washed with ~10 column volumes of loading buffer (~pH8 Tris Buffer, 0.1M NaCl). The sample was applied to the column, reapplied, and was washed with loading buffer. The sample was then eluted in 3CVs of elution buffer (~pH8 Tris Buffer, 2M NaCl), and desalted with a commercial PD-10 column (GE Healthcare, Chicago, IL).

### Purification and Analysis of Heparan Sulfate GAGs Disaccharides

Solutions of the isolated GAG material from pancreas were digested with 1 µL each of Heparinases I–II–III (New England Biolabs, Rowley, MA) to break down Heparin/heparan sulfate GAGs into disaccharides. The enzymatic products were then separated with SAX-HPLC (4.6 × 250 mm Waters Spherisorb analytical column with 5 μm particle at 1.0 ml/min flow rate) coupled to fluorescence detection *via* postcolumn derivatization (43, 44, 46, 47). Assignments were made based on comparison to a separation of known HS disaccharide standards (Dextra Labs, Reading, UK).

### Islet Isolation and *Ex-Vivo* Islet Glucose Stimulated Insulin Secretion (GSIS)

Islets were isolated from P21 using the standard ductal inflation technique (14, 48). Islet GSIS was evaluated using the Biorep Perifusion System (Biorep, Miami Lakes, FL) (49). After 24-hours of recovery in RPMI media, 30 size-matched islets were loaded into a perifusion chamber. Islets were perifused with Krebs buffer containing 2.5 mmol/L glucose for 20 minutes, followed by 15 mmol/L glucose for 40 minutes at a rate of 120 ul/min. Insulin concentrations of perifusion media were measured using Mercodia Mouse Insulin Elisa kit (10-1247-01, Mercodia, Uppsala, Sweden) and normalized to total DNA in each sample.

### Immunohistochemistry and β-cell Area Measurement

Pancreata of offspring were fixed and sectioned into 5 um slices. A total of five sections per animal were analyzed for β-cell area. Pancreatic sections were processed, stained, and analyzed as previously described (14). Immunohistochemistry was performed using anti-insulin antibody (Agilent, Cat# IR002, RRID: AB_2800361, TX, USA). Images of whole pancreas were obtained using Axio-Scan Z1 inverted microscope, and the percent area of β-cell within each section was then calculated as published (14).

### Statistics

All results were represented as mean ± SEM. Percent changes in fat:lean ratio and GTT AUC were calculated by normalizing MatOb offspring data to the mean of sex-matched controls. For single time-point measurement, the difference between the two groups were assessed using the Student’s two-tailed t-test unless stated otherwise. One-way ANOVA test followed by Bonferroni multiple comparison tests correction were performed to assess difference between multiple groups (offspring GTT/ITT AUC, offspring body compositions). For repeated measures (glucose level during GTT, ITT, GSIS), two-way ANOVA tests followed by Bonferroni multiple comparison tests correction were performed to assess the difference between two groups. Results were defined as statistically different when p<0.05.

## Results

### Phenotype of Female Dams on Western Diet

**Figure 1:**
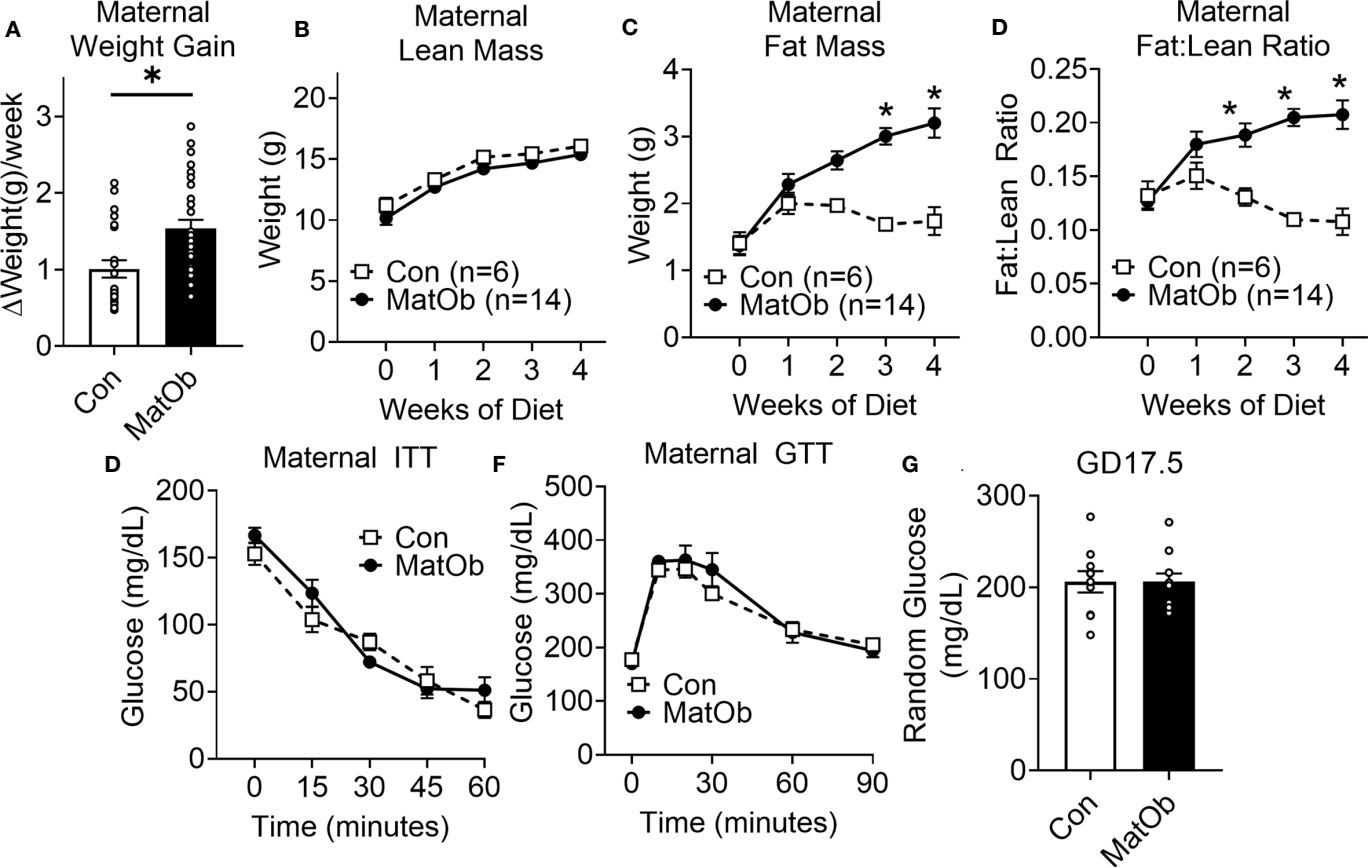
Female dams had increased body adiposity and unchanged glucose and insulin tolerance prior to mating **(A)** Average maternal weight gain per week during western style diet (n= 22-30/group). **(B–D)** EchoMRI was performed on week 0, 1, 2, 3, and 4 on western diet to measure maternal lean weight, fat weight, and fat:lean ratio (n=6-14/group). Prior to mating after completing 4 weeks of diet, female dams had unchanged **(E)** ITT (n=6-9/group) and **(F)** GTT (n=8/group) **(G)** Maternal glucose level at E17.5 (n=13-14/group) (*p < 0.05).

Compared to the control female mice on regular chow (Con), female mice fed a western diet (MatOb) gained significantly more weight (**Figure 1A**, Con: 1.01 ± 0.11 vs. MatOb: 1.54 ± 0.11 g/week, p<0.01). Using EchoMRI, we found that the total body lean mass was similar in between two groups (**Figure 1B**), while the total body fat mass (**Figure 1C**, p=0.054 at week 2; p<0.001 at week 3 and week 4 of diet) and fat:lean ratio (**Figure 1D**, p<0.01 at week 2; p<0.001 at week 3 and week 4 of diet) was significantly increased in MatOb female dams by the second week of the diet. These results indicate that the increase in maternal weight gain is secondary to the increase in total body fat mass. Prior to mating, MatOb female dams showed no difference in insulin (**Figure 1E**) and glucose tolerance (**Figure 1F**). At gestation day 17.5 (GD17.5), MatOb dams had random glucose levels comparable to Con dams (**Figure 1G**).

### P21 Offspring Born to Obese Dams Had a Similar Extent of Increase in Body Adiposity, Unchanged Insulin Tolerance, and Exhibited Sex-Differences in Glucose Tolerance

**Figure 2:**
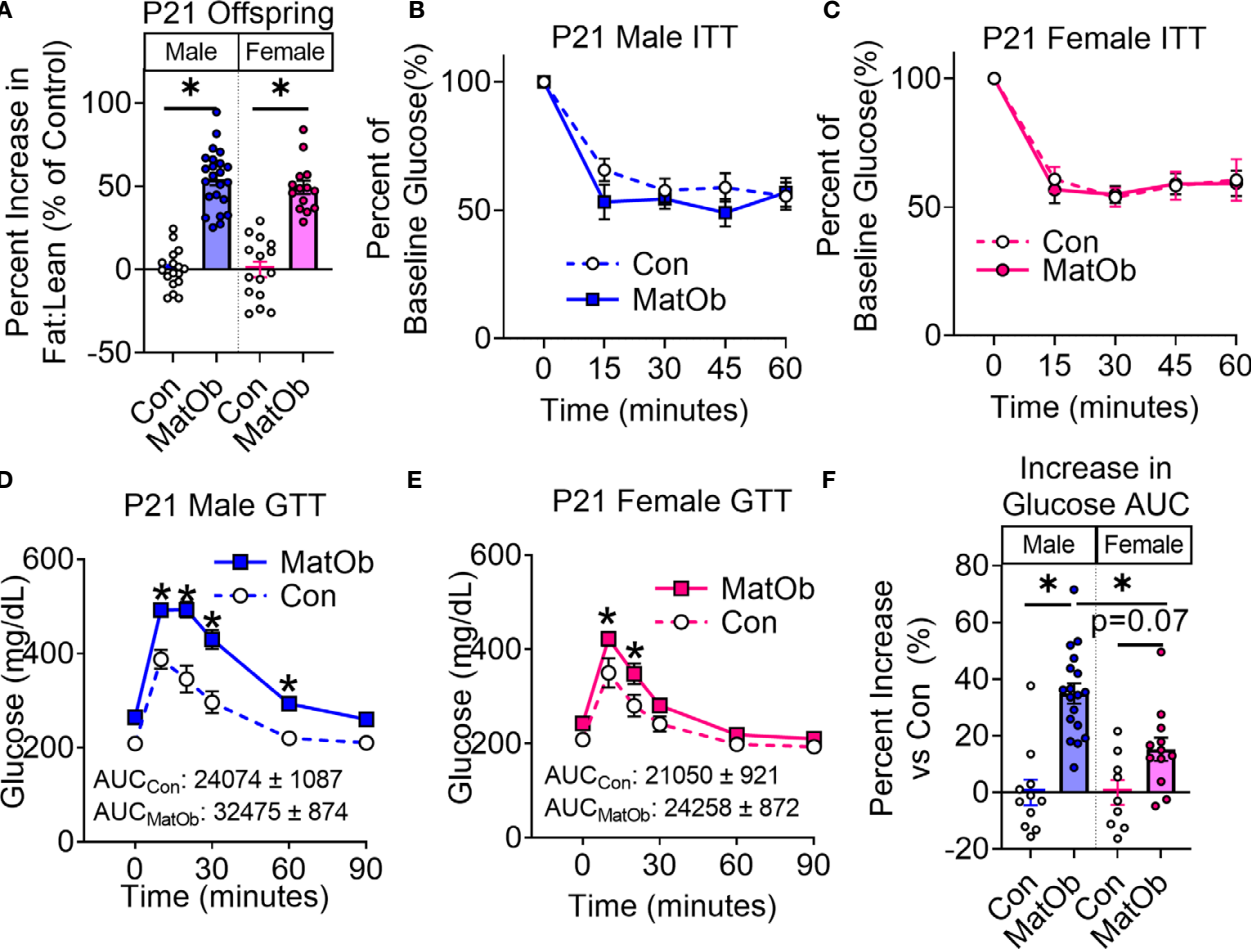
P21 Offspring of obese dams (MatOB) had similar extent of increase body adiposity and unchanged ITT, but exhibited sex-differences in glucose intolerance. **(A)** Percentage changes of fat:lean ratio (normalized to same-sex controls) showing increased total body adiposity in both male and female MatOB offspring (n=15-23/group). Insulin tolerance was unchanged in P21 **(B)** male (n=8-10/group) and **(C)** female offspring (n=9-10/group). **(D)** P21 male MatOb offspring have significantly increase in glucose excursion curve during glucose tolerance testing (n=11-18/group), while **(E)** P21 female MatOb offspring had lesser extent of glucose intolerance (n=9-12/group). **(F)** Percent increase in GTT glucose AUC showing male MatOb offspring have significantly decreased glucose tolerance. Experimental animals originated from at least 5 litters. *p < 0.05.

Compared to same-sex control weanling born to chow-fed dams (Con), both 21-day-old male and female weanling of obese dams (MatOb) had unchanged body weight and lean mass, and exhibited higher total body fat mass and fat:lean ratio (**Supplemental Figure 1**). When normalized to same-sex controls, the percent increase in the fat:lean ratio was similar between male and female MatOb offspring (**Figure 2A**, Con male vs. MatOb male p<0.001, Con female vs. MatOb female p<0.001). We next evaluated the insulin tolerance and glucose tolerance of MatOb offspring, as increased body adiposity is associated with decreased insulin sensitivity and impaired glucose tolerance (50). During insulin tolerance testing (ITT), we found that both male and female MatOb offspring had a similar response to insulin stimulation compared to same-sex controls (**Figures 2B, C**). Both male and female MatOb offspring had elevated glucose levels during GTT (**Figures 2D, E**), but interestingly the extent of glucose intolerance was more prominent in male MatOb offspring (**Figure 2D**: Con male vs MatOb male p<0.001 at 10 min, 20 min, and 30 min, p<0.01 at 60 min; **Figure 2E**: Con female vs MatOb female p<0.01 at 10 min, p<0.05 at 20 min). Compared to male Con offspring, male MatOb offspring had a 35% increase (8401 ± 874 mg/dL*min) in glucose AUC (**Figure 2F**, p<0.0001). In contrast, female MatOb offspring demonstrated only a 15% non-significant increase (3208 ± 872 mg/dL*min) in GTT glucose AUC compared to Con female (**Figure 2F**).

### P21 Male MatOb Offspring Had Lower Islet Insulin Secretion and Pancreatic HSG, While Female MatOb With Higher Pancreatic HSG Had Unchanged Insulin Secretion

**Figure 3:**
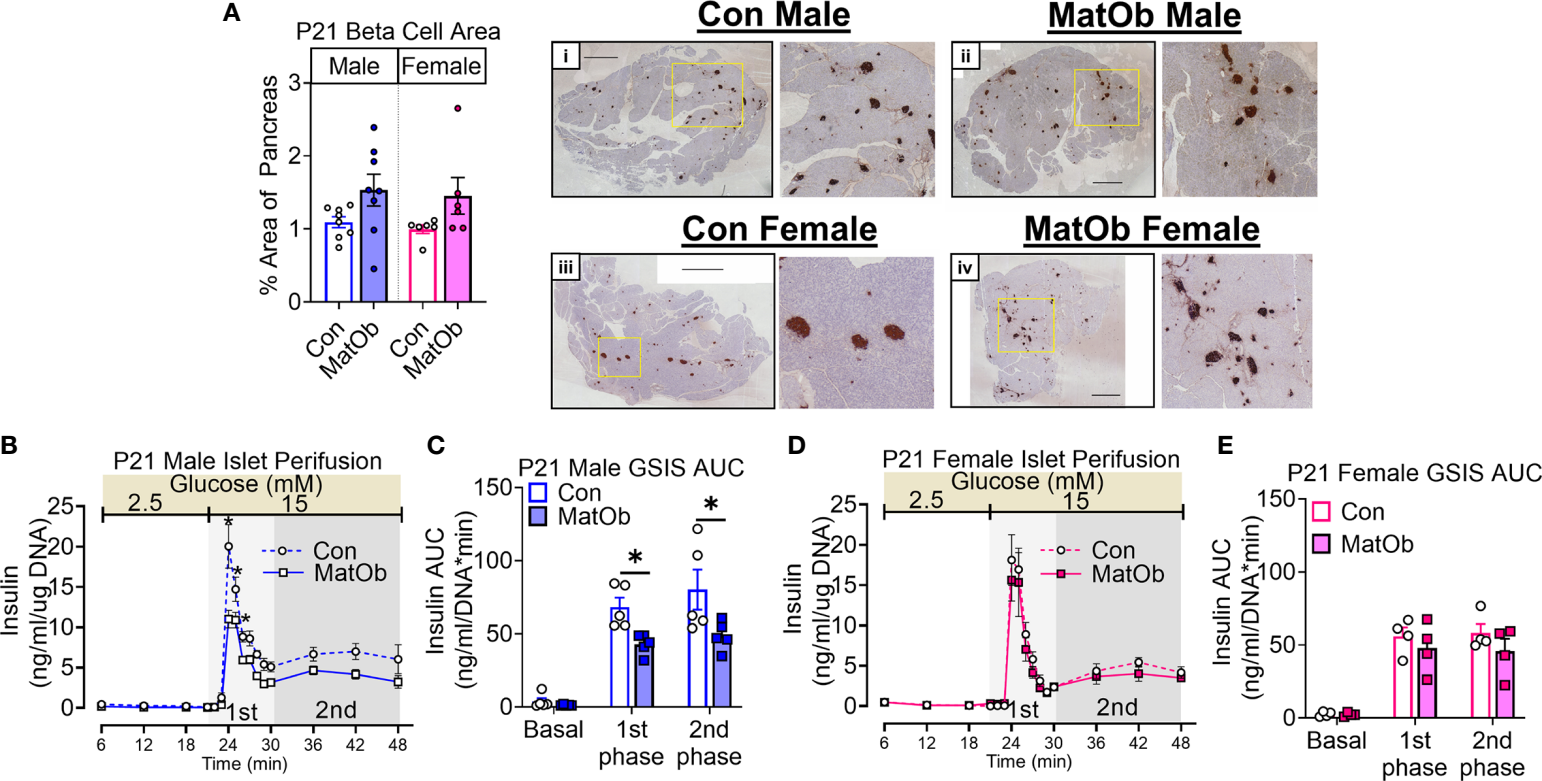
P21 male MatOb offspring have decreased islet insulin secretion. **(A)** β cell area (n = 5 males, 7 females/group) was unchanged in both male and female MatOb offspring at P21. Representative IHC images of whole pancreatic sections with insulin staining (dark brown) and hematoxylin counterstain (Scale bar = 1000um) from (i) Con male, (ii) MatOb male, (iii) Con female, and (iv) MatOb female. Islet perifusion studies showing **(B, C)** decreased glucose-induced insulin secretion in P21 MatOb male offspring (n=5/group), and **(D, E)** unchanged glucose-induced insulin secretion in P21 MatOb female offspring (n=4/group). Experimental animals were originated from at least 3 separate litters (*p < 0.05).

**Figure 4:**
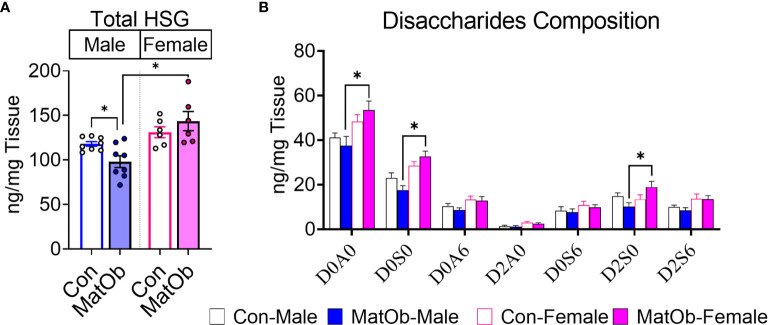
P21 MatOb offspring have sex-specific changes in total pancreatic heparan sulfate glycosaminoglycan (HSG). **(A)** Total pancreatic HSG in male MatOb offspring was decreased, while female MatOb offspring appeared to have the significantly higher HSG compared to male MatOb offspring (n=6-8/group). **(B)** HSG disaccharide composition analysis showed P21 female MatOb offspring had significantly increase D0A0 and D0S0 disaccharides (n=6-8/group; disaccharide structure code letters/numbers: D: Δ4,5-unsaturated uronic acid, A: N=Acetylglucosamine, S: N-Sulfoglucosamine, 0: no sulfation,2: 2-O-sulfation; 6: 6-O-sulfation). Experimental animals were originated from at least 3 separate litters (*p < 0.05).

The difference in male and female MatOb offspring glucose intolerance indicates the possibility of sex-specific differences in pancreatic islet insulin secretion at P21, which is determined by total islet β cell mass and islet insulin secretion. At P21, there was no significant difference in MatOb offspring β cell area, indicating that β cell mass was not altered (**Figure 3A**). We further evaluated glucose-induced insulin secretion in isolated islets and found that when compared to sex-matched controls, male MatOb offspring had ~35% decrease in first- and second-phase insulin secretion (**Figure 3B** p<0.001, p<0.01, and p<0.05 at time 24, 25, 26 min respectively, **Figure 3C** p<0.05 for both 1^st^ and 2^nd^ phase). In contrast, glucose-induced insulin secretion was unchanged in female MatOb offspring (**Figures 3D, E**). We next evaluated the heparan sulfate glycosaminoglycan abundance and the disaccharide composition in Con and MatOb offspring. Compared to sex-matched controls, we found that the total pancreatic HSG was decreased in P21 male MatOb offspring (**Figure 4A**, Con male vs. MatOb male p<0.05). Interestingly, we also observed that female MatOb offspring had significantly higher pancreatic HSG compared to MatOb male offspring (MatOb female vs MatOb male p<0.05). This increase is secondary to an increase in two non-sulfated heparan sulfate disaccharides (MatOb male vs MatOb female D0A0 p<0.05, and D0S0 p<0.001) and a sulfated heparan sulfate disaccharide (D2S0, p<0.05) (**Figure 4B**).

### 8-Week Old MatOb Offspring Had Higher Adiposity, and Persistent Sex-Differences in Glucose Tolerance

**Figure 5:**
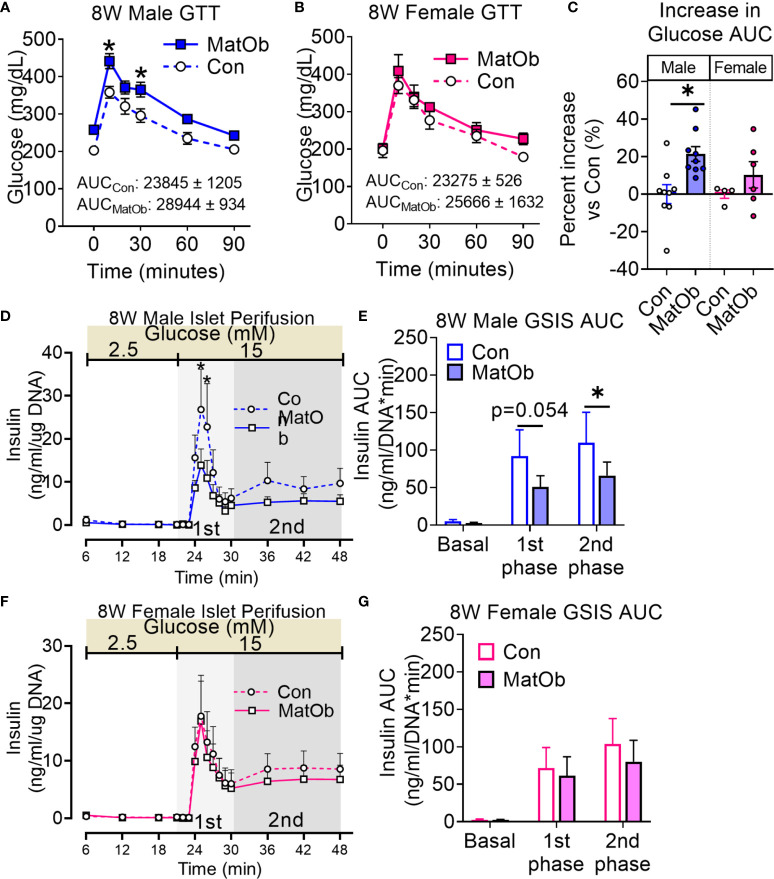
8W MatOb offspring continued to exhibit sex-specific difference in glucose intolerance and islet insulin secretion. **(A)** 8W male GTT (n=9/group), **(B)** 8W female GTT (n=4-6/group), **(C)** Percent increase in GTT glucose AUC normalized to sex-matched controls. Islet perifusion studies showing **(D, E)** persistently decreased glucose-induced insulin secretion in 8W MatOb male offspring (n=4/group), and **(F, G)** unchanged glucose-induced insulin secretion in 8W MatOb female offspring (n=4/group). Experimental animals originated from at least 4 separate litters, *p < 0.05.

We next determined if the earlier changes in metabolic phenotype and islet insulin secretion would persist after weaning to a regular chow diet. After weaning to a regular chow diet, 8-week old (8W) male MatOb offspring were found to have a similar body weight and lean mass, while female MatOb offspring were found to have a lower total body weight and lean mass (**Supplemental Figure 2**). Similar to findings on P21, both male and female 8W MatOb offspring were found to have similar extent of increase in fat:lean ratio (**Supplemental Figure 2**). The sex-differences in glucose tolerance persisted in MatOb offspring. Compared to sex-matched controls, 8W male MatOb remained more glucose intolerant (**Figure 5A**, p<0.01 at time 10 and 30 min, **Figure 5C** Con male vs MatOb male p<0.01), but female MatOb had no significant difference in glucose tolerance (**Figures 5B, C**). Lastly, 8W male MatOb offspring continued to have lower islet insulin secretion (**Figure 5D**, p<0.001 at time 25 and 26 min, **Figure 5E**, 1^st^ phase p=0.0548, 2^nd^ phase p<0.05), while female MatOb offspring continued to have unchanged islet insulin secretion (**Figures 5F, G**). Collectively, these results demonstrated the impact of maternal obesity on sex-differences in glucose tolerance in offspring, secondary to islet insulin secretory dysfunction. Importantly, we also demonstrated the sex-differences in pancreatic heparan sulfate glycosaminoglycan and the early onset of glucose intolerance and islet dysfunction prior to weaning.

## Discussions

Human offspring born to obese mothers suffer higher risks of obesity (5–8, 51) and insulin resistance (10–12). The combination of increased adiposity and insulin resistance induce metabolic stress to pancreatic islets (52). To prevent hyperglycemia and progression to diabetes, pancreatic β cells compensate through mass expansion and an increase in insulin secretion (53, 54). In contrast, the increased risks of type 2 diabetes in offspring of obese mothers reflects the failure of the pancreatic β cell to compensate during a state of increased insulin demand.

In this paper, we first validated the negative impact of maternal obesity on overall offspring metabolic health at weaning. We used a diet-induced maternal obesity model to depict pre-pregnancy without the development of overt insulin resistance and gestational diabetes. We found that P21 offspring born to obese dams have increased adiposity, which is consistent with previously reported animal studies (55) and is similar to the features of human offspring of obese mothers (12, 24, 56). While insulin resistance has been reported in offspring born to obese mothers as early as the newborn period (25), we did not observe any significant differences in the response of MatOb offspring to insulin stimulation during insulin tolerance testing. Such a discrepancy may be expected, as de Fante et al. demonstrated in a similar diet-induced maternal obesity model. Their work supported that a postnatal high-fat diet challenge is required to accentuate insulin resistance in offspring mice born to obese dams (57). We further demonstrated that offspring of obese dams developed sex-differences in glucose intolerance and islet insulin secretion. The development of sex-specific differences in glucose intolerance prompted the detailed evaluation of pancreatic β cell function, which is determined by total β cell mass and insulin secretion. Overall, MatOb offspring did not have statistically significant changes in β cell area, suggesting that impaired β cell mass expansion was not the primary mechanism leading to glucose intolerance. On the contrary, we found that male MatOb had decreased insulin secretion in isolated islets. This finding supported the premise that decreased glucose tolerance in male MatOb is secondary to the decrease in intrinsic islet glucose-induced insulin secretion. Notably, this is the first paper that reported the presence of decreased islet insulin secretion in MatOb offspring as early as P21.

The sex-differences in glucose intolerance and islet insulin secretion persisted in 8-week-old MatOb offspring, suggesting the long-lasting programming effect of maternal obesity. One limitation within our study is that the estrous cycle was not evaluated to minimize stress prior to metabolic studies. We reasoned that housing age-matched Con and MatOb female offspring in the same cage with no exposure to male animals would keep these female in anestrus phase (58, 59). Secondly, the sex-differences observed in MatOb offspring at 8-weeks may indicate the potential contributions of sex chromosomes and sex hormones (60, 61) on other tissues/organ systems, such as the hypothalamus and adipose tissue, that could negatively impact offspring appetite or adipogenesis (62). However, we reasoned that these are unlikely in our model, as there was a lack of sex-differences in the increase in total body adiposity and the response to insulin stimulation in male and female MatOb offspring compared to the sex-matched Con offspring weaned to same cage. Additionally, the sex differences in islet insulin secretory dysfunction were present during *ex-vivo* GSIS, where islets were cultured using the same media and *ex-vivo* GSIS were performed with serum-free buffers containing no sex-hormones.

Another novel observation of this study was the pattern of pancreatic HSG changes that is consistent with the sex-differences in offspring glucose tolerance and islet insulin secretion. The biosynthesis of HSG is a complex process regulated by over 25 enzymes, where initially a tetrasaccharide linkage region is formed at the serine residue of the heparan sulfate core protein (63, 64). This is followed by the chain initiation and elongation process. This process involves the addition of repeating disaccharide units, and finally chain modification that involves processes such as N-deacetylation, N- or O-sulfation, and C5 epimerization of different disaccharide residues. Disruption in any of these steps has the potential to alter the structure of HSG chains that determines its biological functions. As a specific example of pancreatic islet insulin secretion function, EXTL3 glycosyltransferase knock outs within the β-cell results in a lower HSG and islet insulin secretion (39). In our study, the male MatOb offspring with a decrease in pancreatic HSG had more apparent glucose intolerance and a significant decrease in islet insulin secretion. In contrast, female MatOb offspring with higher pancreatic HSG had unchanged islet insulin secretion. The changes in total mass of pancreatic HSG and three disaccharides (D0A0, D0S0, D2S0), along with and an unchanged compositional makeup (percentage w/w of disaccharides to total HSG, **Supplemental Table 2**) indicate a decrease in the length of the HSG chain. Since sexual maturation occurs after P21 in mice (peripubertal P28-P40) (65), the sex-differences in pancreatic HSG and islet insulin secretion observed as early as P21 are unlikely driven by sex hormones. Another limitation of this study is that the measurement of HSG was performed on pancreatic tissue, which may not reflect the levels of HSG within islets. However, immunohistology studies from two independent groups showed that pancreatic islets have higher HSG compared to surrounding acinar tissues (40, 66, 67). Therefore, we postulate that the magnitude of differences observed in our HSG analysis may have been attenuated. The final limitation of this study is that molecular pathways leading to altered HSG, and the subsequent impact on downstream signals were not explored. HSG has been reported to regulate cellular functions primarily through interactions with other proteins, where over 200 proteins were predicted to interact with HSG, and the majority (66%) of these proteins are expressed intracellularly (68). While the precise pathway leading to islet dysfunction in MatOb was not identified, our study still demonstrates the sex-specific impact of maternal obesity exposure on offspring glucose tolerance and islet dysfunction, particularly the importance of evaluating islet insulin secretion at a younger age. Future studies are warranted to characterize the temporal changes of pancreatic HSG in MatOb offspring, and further delineate the molecular mechanisms leading to islet dysfunction in MatOb offspring.

In summary, our findings demonstrate that MatOb offspring develop sex-specific differences in islet dysfunction and pancreatic heparan sulfate glycosaminoglycan. These results will spur a new research direction investigating the mechanistic role of HSG in the patterning of islet dysfunction in the offspring of obese mothers.

## Data Availability Statement

The raw data supporting the conclusions of this article will be made available by the authors, without undue reservation.

## Ethics Statement

The animal study was reviewed and approved by Indiana University School of Medicine Institutional Animal Care and Use Committee.

## Author Contributions

JC and KK contributed to the conception and design of the study. JC, JJ, RB, KO, and SA-H designed and performed experiments. JC, SA-H, PA, and KK performed data analysis, and all authors participated in the results interpretation. CD and JC wrote the first draft of manuscript. All authors contributed to the article and approved the submitted version.

## Funding

This project was funded by the Riley Children’s Foundation Physician Scientists Scholar Award (KK), Showalter Research Trust Fund (EPAR1143 to KK), and Center for Diabetes & Metabolic Diseases Pilot & Feasibility Award (P30DK097512, KK). This research was supported in part by the National Institutes of Health (R24GM137782-01 PA) at the Complex Carbohydrate Research Center.

## Conflict of Interest

The authors declare that the research was conducted in the absence of any commercial or financial relationships that could be construed as a potential conflict of interest.

## Supplementary Material

Supplemental Figure 1 P21 offspring **(A)** total body weight, **(B)** lean mass, **(C)** fat mass, and **(D)** Fat:lean ratio. (n=15-23/group, corresponding to **Figure 1A**, *p < 0.05) Experimental animals originated from at least 4 separate litters.

Supplemental Figure 2 At 8 weeks, **(A, B)** total body weight was unchanged and lean mass was decreased in male MatOb offspring, while female MatOb offspring had lower total body weight and lean mass. **(C)** Percentage changes in fat:lean ratio (normalized to same-sex controls) shows both male and female MatOB offspring continued to have similar extent of increase in fat:lean ratio. (n=4-13/group, *p < 0.05) Experimental animals were originated from at least 4 separate litters.

Supplemental Table 1 Table 1 showing composition of macronutrients in % weight.

Supplemental Table 2 Table 2 showing percentage (%w/w) of HSG Disaccharides in pancreas collected from P21 offspring measured using HPLC.
