# Supplementary figures and images for: Offspring of Obese Dams Exhibit Sex-Differences in Pancreatic Heparan Sulfate Glycosaminoglycans and Islet Insulin Secretion
Source: Front Endocrinol (Lausanne). 2021 May 24;12:658439. doi: 10.3389/fendo.2021.658439 (PMC8181410; doi:10.3389/fendo.2021.658439)

# Supplemental Figure 1

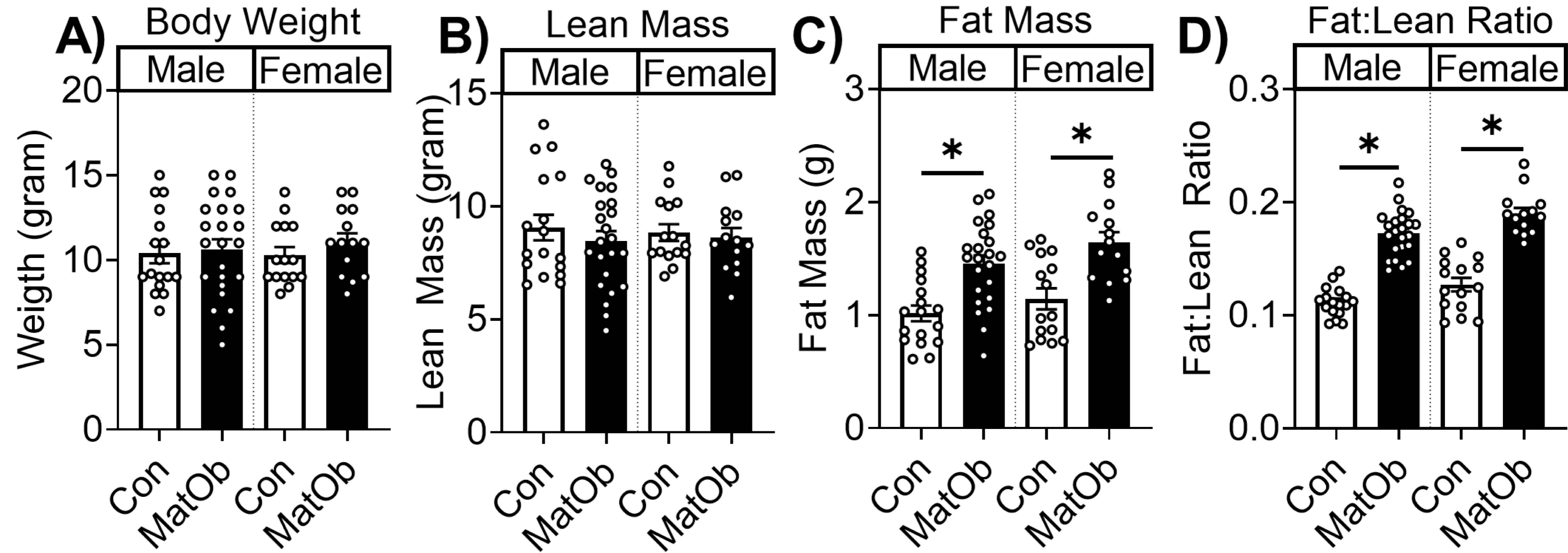

# Supplemental Figure 2

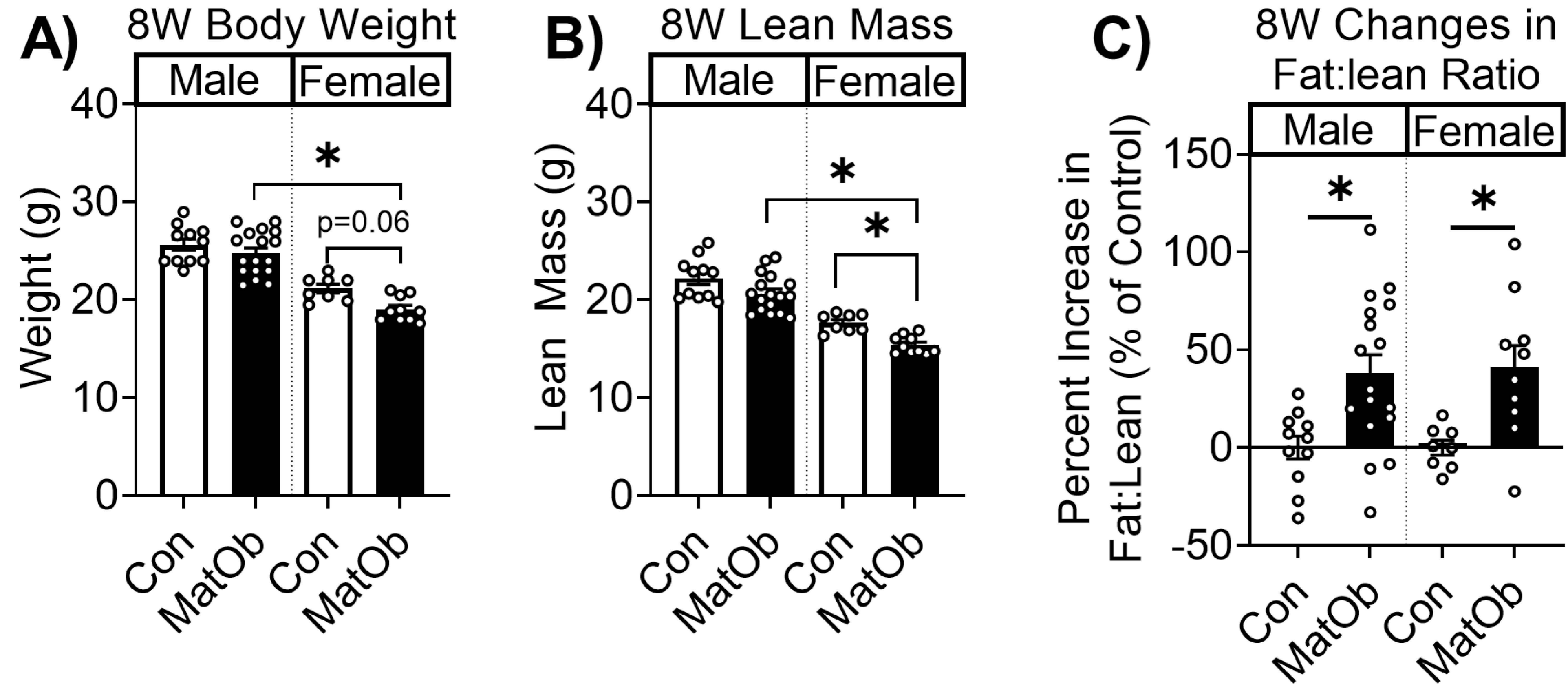

Supplement: Supplemental Figure 1 — P21 offspring (A) total body weight, (B) lean mass, (C) fat mass, and (D) Fat:lean ratio. (n=15-23/group, corresponding to Figure 1A , *p < 0.05) Experimental animals originated from at least 4 separate litters. [file Image_1.pdf]
