# Supplementary material for: Offspring of Obese Dams Exhibit Sex-Differences in Pancreatic Heparan Sulfate Glycosaminoglycans and Islet Insulin Secretion
Source: Front Endocrinol (Lausanne). 2021 May 24;12:658439. doi: 10.3389/fendo.2021.658439 (PMC8181410; doi:10.3389/fendo.2021.658439)
Supplement: Supplemental Table 1 — Table 1 showing composition of macronutrients in % weight. [file Table_1.docx]

**Supplemental Table 1**

Macronutrients of animal diets

|  |  | Chow (2018 Teklad global)  (%Weight) |  | Western Style Diet (TD.88137)  (%Weight) |
| --- | --- | --- | --- | --- |
| Fat |  | 7 |  | 21.2 |
| Carbohydrate |  | 44.2 |  | 48.5 |
| Protein |  | 18.6 |  | 17.3 |
| Fiber |  | 3.5 |  | 5 |

**Supplemental Table 2**

Percentage (% w/w) of HSG Disaccharides

|  | Con-Male | MatOb-Male | Con-Female | MatOb-Female |
| --- | --- | --- | --- | --- |
| HS |  |  |  |  |
| D0A0 | 38.4 ± 0.8 | 40.2 ± 1.1 | 36.8 ± 0.8 | 36.8 ± 0.8 |
| D0S0 | 20.7 ± 1.1 | 20.3 ± 1.2 | 22.2 ± 1.7 | 23.0 ± 1.6 |
| D0A6 | 9.6 ± 1.1 | 9.3 ± 1.1 | 10.2 ± 0.9 | 8.7 ± 0.7 |
| D2A0 | 1.0 ± 0.4 | 1.1 ± 0.5 | 2.3 ± 0.4 | 1.8 ± 0.3 |
| D0S6 | 7.4 ± 1.4 | 8.1 ± 1.4 | 8.7 ± 1.7 | 7.2 ± 1.1 |
| D2S0 | 13.7 ± 1.3 | 11.7 ± 1.3 | 10.0 ± 1.3 | 12.8 ± 1.1 |
| D2A6 | 0 ± 0 | 0 ± 0 | 0 ± 0 | 0 ± 0 |
| D2S6 | 9.0 ± 0.5 | 8.8 ± 0.6 | 10.2 ± 1.3 | 9.3 ± 1.1 |
